# Threshold-Based User-Assisted Cooperative Relaying in Beamspace Massive MIMO NOMA Systems

**DOI:** 10.3390/s22197445

**Published:** 2022-09-30

**Authors:** David Alimo, Masanori Hamamura, Saifur Rahman Sabuj

**Affiliations:** 1 Graduate School of Engineering, Kochi University of Technology, 185 Miyanokuchi, Tosayamada, Kami City 782-8502, Japan; 2 School of Information, Kochi University of Technology, 185 Miyanokuchi, Tosayamada, Kami City 782-8502, Japan; 3 Department of Electronic Engineering, Hanabat National University, Daejeon 34158, Korea; 4 Department of Electrical and Electronic Engineering, Brac University, Dhaka 1212, Bangladesh

**Keywords:** beamspace, massive multiple-input multiple-output (mMIMO), cooperative relaying, non-orthogonal multiple access (NOMA)

## Abstract

The incorporation of user-assisted cooperative relaying into beamspace massive multiple-input multiple-output (mMIMO) non-orthogonal multiple access (NOMA) system can extend the coverage area and improve the spectral and energy efficiency for millimeter wave (mmWave) communications when a dynamic cluster of mobile user terminals (MUTs) is formed within a beam. We propose threshold-based user-assisted cooperative relaying into a beamspace mMIMO NOMA system in a downlink scenario. Specifically, the intermediate MUTs between the next-generation base station (gNB) and the cell-edge MUT become relaying MUTs after the successful decoding of the signal of the cell-edge MUT only when they meet the predetermined signal-to-interference plus noise ratio (SINR) threshold. A zero forcing (ZF) precoder and iterative power allocation are used to minimize both inter- and intra-beam interferences to maximize the system sum rate. We then evaluate the performance of this system in a delay-intolerant cell-edge MUT scenario. Moreover, the outage probability of the cell-edge MUT of the proposed scheme is investigated and an analytic expression is derived. Simulation results confirm that the proposed threshold-based user-assisted cooperative relaying beamspace mMIMO NOMA system outperforms the user-assisted cooperative relaying in beamspace mMIMO NOMA, beamspace MIMO-NOMA, and beamspace MIMO orthogonal multiple access (OMA) systems in terms of spectrum efficiency, energy efficiency, and outage probability.

## 1. Introduction

With the roll-out of 5G mobile networks and the emergence of new applications, significant data growth is expected in the next few years. It is predicted that by 2030, the total mobile data traffic will increase to 5 zettabytes (ZB) per month and the number of mobile user terminals (MUTs) will be more than 125 billion units [1]. To meet this demand, industry and academia must devise innovative technologies that can meet these requirements in smarter and innovative ways [2]. To achieve higher capacity, low latency, and low power consumption, it will be necessary to exploit the advantages of massive multiple-input multiple-output (mMIMO) and non-orthogonal multiple access (NOMA) technologies [3]. Millimeter wave (mmWave) technology is suitable for implementing mMIMO systems [2,3,4]. It has a large number of unused frequencies and a small wavelength and has, thus, attracted considerable attention from both industry and academia [2,3,4,5]. It allows many antenna elements to fit in a small physical space and can improve the spectral efficiency [2,3,4,6]. However, its high transceiver complexity and energy consumption make its practical application difficult to realize [2,3,4,6,7,8,9,10,11,12,13]. Because each antenna in a MIMO system typically requires one dedicated radio-frequency (RF) block (with subunits including an amplifier, filter, mixer, oscillator, and converter), the immense hardware expense and energy usage due to a large number of RF blocks in mmWave mMIMO systems appear to be inevitable [2,3,4,6,7,8,9,10,11,12,13].

The proposal of beamspace mMIMO systems using a lens antenna array, hybrid precoding, and beam selection have considerably reduced the number of RF blocks in mmWave mMIMO systems with no apparent performance loss [2,3,4,6,7,8,9,10,11,12,13,14,15], but the maximum number of MUTs that can be supported at the same time and frequency resources cannot surpass the number of available RF blocks [2,3,4,7,9,13,16]. To overcome this limitation, NOMA has been incorporated into beamspace mMIMO systems to allow multiple MUTs to be served simultaneously in the same RF block [1,2,3,4,6,7,8,9,10,11,12,13,17]. NOMA has been demonstrated to perform better than orthogonal multiple access (OMA) schemes in terms of spectral and energy efficiency, outage probability, and other factors, making it an excellent solution for 5G networks and beyond [1,4,7,18]. In NOMA, a power domain is used to multiplex multiple MUTs instead of using conventional OMA techniques (e.g., time/frequency/code division multiple access) [19]. Different MUTs are given different power levels with the same time, frequency, and code resources [1,2,3,4]. This allows the superposition of signals with different power levels at the next-generation base station (gNB) and successive interference cancellation (SIC) on the MUT (i.e., receiver) side to eliminate multi-MUT interference [1,2,4]. As a result, it is possible to accommodate more MUTs at the expense of introducing manageable inter-MUT interference [1,2,4,20,21,22,23]. Therefore, by incorporating NOMA into mmWave beamspace mMIMO systems, the spectral efficiency can be increased [1,2,4]. Even though mmWave communication is suitable for NOMA implementation, it is limited to short-distance communications [3].

### 1.1. Related Works

Device-to-device (D2D) and relay-aided cooperative communications have attracted attention and are considered a promising new paradigm for current and future wireless networks [24,25]. In D2D communications, two adjacent MUTs in a cellular network can transmit signal directly without them passing through the gNB [24,25]. Subsequently, D2D communications can enhance the spectral efficiency, throughput, and energy efficiency, and reduce the delay [24,25]. In [26], a D2D communication where D2D transmitters relay signals to cellular MUTs was investigated in terms of outage probability and average feasible rate, and the total feasible rate was maximized using an optimal spectrum and power allocation strategy in a cellular network with multiple D2D pairs. Another study [27] analyzed the performance of multi-hop D2D communications using the shortest path algorithm in the presence of co-channel interference from other D2D communication pairs and conventional cellular MUTs in both uplink and downlink in terms of outage probability and concluded that although the D2D links are reliable, they can severely degrade the performance of conventional cellular MUTs.

Relay-aided cooperative communication incorporating NOMA is another promising technique that can enhance the spectrum efficiency and the coverage in areas with poor coverage [3,25,28,29,30,31,32,33,34,35,36,37]. Relay-aided communication can be enabled by several deployment modes, encompassing a fixed dedicated relay, mobile dedicated relay, and user-assisted relay enabled by D2D communications [28,29]. A NOMA-based cooperative relaying system (CRS) using a dedicated relay was considered in [34,35,36], where the dedicated relay forwards the signal from the gNB to the destination or vice versa. To be specific, a novel full-duplex relay transmission mode in a dedicated decode-and-forward relay was proposed in [34], in an uplink scenario where SIC and self-interference cancellation (SC) were utilized to decode the symbols of two MUTs at the relay prior to transmitting the superimposed signal to the gNB. The proposed full-duplex NOMA CRS outperforms the half-duplex NOMA/OMA CRS in terms of ergodic sum rate and outage probability. An approximation method using the Gauss–Chebyshev method to calculate the average feasible rate was derived for a NOMA-based CRS over the Rician fading channel [35], and it was revealed that the derived analytical results matched the Monte Carlo simulations and that the NOMA-based CRS achieved a higher achievable rate than the traditional CRS. Additionally, a NOMA-based amplify-and-forward CRS with a novel detection strategy was proposed in [36] and verified that the relaying strategy can obtain full cooperative diversity. Despite the benefits, relay-aided communications deployment using fixed or mobile dedicated relays requires enormous power consumption and an extremely high cost to mobile network operators [35,38]. Hence, user-assisted cooperative relaying becomes the potential candidate to provide flexibility in extending the coverage area of mobile networks.

User-assisted CRS have been investigated in various research works [3,30,39]. Elkotby et al. [25] used stochastic geometry to analyze the performance of partial decode-and-forward uplink user-assisted relaying in cellular networks in terms of average rate and cooperation probability and showed that user-assisted relaying can significantly improve the per-MUT transmission rate despite increased inter-cell interference. In a similar study, Liau et al. [30] proposed a novel power splitting algorithm and used a pair of MUTs near the gNB to decode-and-forward the signal of a far MUT successively in a NOMA-based user-assisted CRS. Two scenarios, including the availability of the non-casual state information at both the source and the relay and exclusively at the source, were evaluated in [40] for a partially cooperative relay broadcast channel with state information. Relay channels and cooperative relay broadcast channels controlled by random parameters were investigated in [41]. It has been demonstrated that, in some situations, decode-and-forward relaying can reach the capacity area when the state information is non-casually known to the transmitter and intermediary nodes. In [3], user-assisted cooperative relaying for mmWave communications using half-duplex decode-and-forward relays was taken into consideration. This technique revealed the advantages of user-assisted cooperative relaying in beamspace mMIMO NOMA in terms of spectrum and energy efficiencies [3]. Despite the benefits in [3], the message intended for the cell-edge MUT within a cluster is divided into a number of symbols equal to the number of intermediate MUTs between the gNB and the destination, and in each time slot, one symbol is relayed to the cell-edge MUT by only one relay, resulting in a time-slot-hungry relaying system. Hence, the fundamental limitation of this system proposed in [3] is that it cannot be applied to a delay-intolerant or low-latency system.

On the other hand, to improve the performance of the user-assisted CRS further in terms of outage probability and throughput, several studies investigated exploiting full/half-duplex MUT relaying in NOMA CRS [19,42,43]. In [19], two cooperative relaying schemes were proposed in a NOMA-based user-assisted CRS, namely, on/off full-duplex and on/off half-duplex, and a mechanism to decide whether cooperative relaying is necessary or not, and analyze the performance in terms of outage probability and throughput. The authors in [42] proposed a novel cooperative user-assisted relaying in NOMA systems, where one MUT is employed as decode-and-forward relay switching between full-duplex and half-duplex operation modes. Closed-form expressions for asymptotic outage probabilities and a delay-limited throughput for two NOMA MUTs were derived [42]. Guo et al. [43] investigated a NOMA-based user-assisted CRS in downlink where near MUTs are viewed as full/half-duplex decode-and-forward relays to support multiple far MUTs. Specifically, the impact of the randomness of MUT locations on the system performance was studied using stochastic geometry and evaluated in terms of outage probability [43]. As future mobile networks will be densely populated [1], deploying user-assisted CRS in future mobile networks will render cellular networks difficult to manage due to high network complexity and interference.

As a result, several works [31,32,33,39,44] proposed threshold-based relaying strategies to reduce the number of relaying MUTs while achieving near optimal performance. In [31], threshold-based selective cooperative NOMA user-assisted relaying was proposed and the closed-form expression of the end-to-end bit error rate was derived. Moreover, the optimal threshold value is analyzed to minimize the bit error probability [31]. Similarly, in [32], ergodic capacity and outage probability for threshold-based selective cooperative NOMA user-assisted CRS were analyzed and closed-form expressions for ergodic capacity and outage probability were derived. In [33], amplify-and-forward relays were separated into two pools: (1) relays with a signal-to-noise ratio (SNR) above a threshold and (2) relays that do not meet this threshold. The gNB randomly selects one relay from the first pool to relay to the MUTs within its partition [33]. This study [33] demonstrated that the transmit power can be significantly reduced by choosing an appropriate selection threshold. Kundu et al. [44] proposed three threshold-based relay selection strategies for decode-and-forward relays to reduce the secrecy outage probability. The authors confirmed that the diversity gain of the secrecy outage probability can be maximized by increasing the number of relays [44]. A multiple-threshold-based relaying strategy was proposed in [39], where the mode of a relay is determined by the number of packets in its buffer and the threshold for each relay is independent. A relay whose number of packets is greater than the threshold is designated as a transmission relay, and from among the transmission relays, the relay with the most packets is selected to forward symbols to the destination [39]. Using asymptotic Markov chain analysis, El-Zahr et al. [39] highlighted the impact of threshold levels on the outage probability, queuing delay, and diversity order. Other studies investigated joint buffer-aided relay selection and power allocation in half-duplex decode-and-forward hybrid NOMA/OMA CRS to maximize the throughput with delay constraint [37]. The threshold-based user-assisted CRS used in [3,32] only took into account one relay and one cell-edge MUT, necessitating performance evaluation in scenarios involving multiple user-assisted relaying. In [33,39,44], only one relay was selected to forward symbols to the cell-edge MUT, and when no relay satisfied the threshold, there was no transmission. Furthermore, even with careful power allocation and buffer size design, the buffer-aided CRS in [37,39] imposes an inevitable delay (i.e., two time slots plus buffer delay), which makes it difficult to implement in a delay-intolerant scenario. The contrast between the proposed method and closely related works in the literature is presented in Table 1.

### 1.2. Contributions

In this study, we propose threshold-based user-assisted cooperative relaying in beamspace mMIMO NOMA for mmWave communications, where the intermediate MUTs with good channel conditions are selected to send symbols intended for the cell-edge MUT within the dynamically grouped cluster to improve the spectral efficiency, energy efficiency, and outage probability. The cellular network complexity can be decreased by utilizing a threshold to select the relay MUTs, making the network manageable with no visible performance loss. The multi-hop user-assisted relaying in [3] can maximize the system sum rate. However, it introduces a high delay or latency. As such, it is not suitable in delay-intolerant or low-latency systems. Additionally, including all intermediate MUTs in cooperative relaying will increase network complexity and make the network unmanageable. Through the use of threshold-based user-assisted relay selection, we are able to compromise between network complexity and system performance. Specifically, the main contributions of this paper are outlined as follows:

- We propose threshold-based user-assisted cooperative relaying in beamspace mMIMO NOMA for mmWave communications to improve the overall system and cell-edge MUT performance with low end-to-end latency. To reduce inter- and intra-beam interferences, a zero forcing (ZF) precoder and iterative power allocation are used.
- We compare the performance of this system, CRS beamspace mMIMO NOMA [3], beamspace MIMO-NOMA [4], and MIMO-OMA in a delay-intolerant scenario (A delay-intolerant system refers to a system in which symbols must be received within a specified time frame.). By selecting relaying MUTs based on the signal-to-interference plus noise ratio (SINR) threshold, the cell-edge MUT can receive its symbols in only two transmission phases while maximizing the received SINR.
- We then derive an analytic expression for the outage probability at the cell-edge MUT. This allows us to analyze the proposed system in terms of outage probability and demonstrate its reliability.
- Numerical results revealed that the proposed system achieves superior performance in terms of spectral and energy efficiency. Moreover, the proposed system showed superior performance to CRS beamspace mMIMO NOMA [3], beamspace MIMO-NOMA [4], and MIMO-OMA systems in terms of the outage probability of the cell-edge MUT.

The rest of this paper is structured as follows. Section 2 provides the system model, which is made up of the network architecture and signal model for the beamspace mMIMO NOMA. Section 3 gives the spectral efficiency and outage probability of the proposed threshold-based user-assisted CRS in beamspace mMIMO NOMA. The simulation parameters and results for the proposed threshold-based user-assisted CRS in beamspace mMIMO NOMA are presented in Section 4. Finally, conclusions are provided in Section 5.

**Notation:** We employ lower-case and upper-case boldface characters $\left(a\;\mathrm{and}\;A\right)$ to indicate vectors and matrices, respectively. ${\left(\cdot \right)}^{-1}$, ${\left(\cdot \right)}^{H}$, and $\mathrm{diag}[p_{1},p_{2},\cdots ,p_{K}]$ denote the inverse and conjugate transpose of a matrix and a diagonal matrix of size K×K, respectively. ${∥\cdot ∥}_{2}$ denotes the $\ell_{2}$-norm and $E\left(\cdot \right)$ denotes the expectation. |B| denotes the number of elements in set B and $A{\left(i,:\right)}_{i\in B}$ denotes a submatrix of A that consists of the *i*th row of A for all i∈B. Finally, $\mathrm{CN}\left(m,v\right)$ denotes the complex Gaussian distribution with mean *m* and variance *v* and $\mathrm{Pr}\left(A\right)$ denotes the probability of the occurrence of event A.

## 2. System Model

### 2.1. Network Architecture

We present a detailed description of the beamspace MIMO and the considered beamspace mMIMO NOMA architectures. The architectures consist of four main functional blocks:

- *Lens antenna array*: used to simultaneously realize the functions of signal emitting and phase shifting [16,45].
- *Selector network*: used to reduce the MIMO dimensions by selecting certain beams because a limited number of effective propagation paths exist in mmWave communications and, thus, the channel power is concentrated in a small number of beams [16,45].
- *RF block*: a transceiver subunit consisting of an amplifier, filter, mixer, oscillator, and analog-to-digital/digital-to-analog (A-D/D-A) converters [16,45].
- *Digital precoder*: used to perform the digital baseband signal processing.

**Figure 1:**
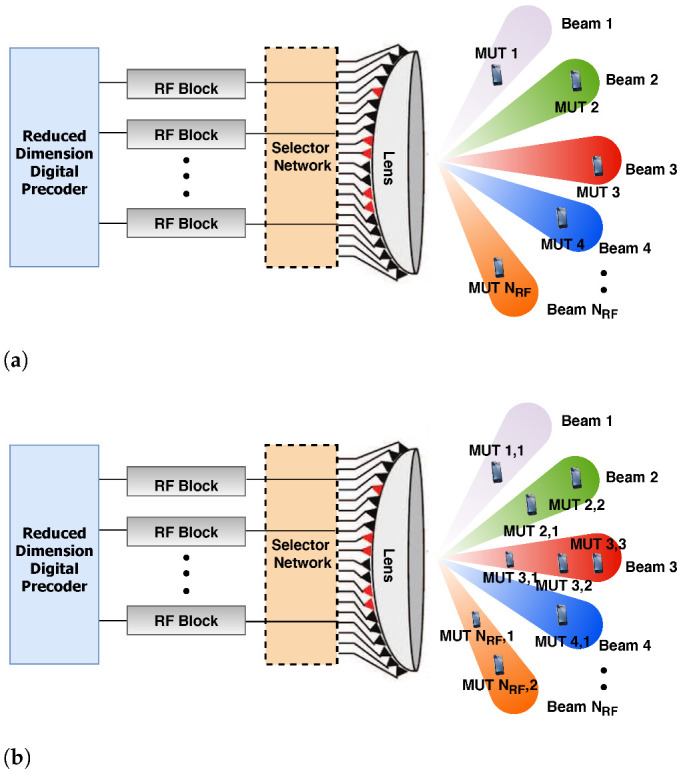
Network architectures: (**a**) Beamspace MIMO. (**b**) Beamspace mMIMO NOMA.

The network architecture considered in this paper consists of a single cell in a downlink mmWave beamspace mMIMO NOMA communication system, where the gNB has *N* antennas and $N_{RF}$ RF blocks [2,4,8,9]. In this architecture, the gNB serves *K* MUTs simultaneously and each MUT is equipped with a single antenna [2,4,8,9]; thus, half-duplex transmission is employed. By using a lens antenna array at the gNB to convert the spatial channel into a sparse beamspace channel, the beamspace MIMO system, shown in Figure 1a, can improve the energy efficiency and reduce the hardware complexity in mmWave MIMO systems [2]. As a consequence, a limited number of beams are required to serve MUTs with no notable performance loss, thus reducing the required number of RF blocks [2,8,9]. However, the number of MUTs that can be supported is limited to one MUT per beam for the same time and frequency resources [2,4]. As such, NOMA was integrated into the beamspace MIMO architecture to overcome this fundamental limit [4]. Using beamspace mMIMO NOMA, as shown in Figure 1b, numerous MUTs can be served simultaneously within each selected beam by leveraging NOMA [2,3,4,35]. Therefore, the total number of supported MUTs can exceed the total number of RF blocks [2,4].

### 2.2. Signal Model

In this paper, we consider the well-known Saleh-Valenzuela channel model for mmWave communications, where the spatial channel vector between the gNB and the *k*th (k=1,2,⋯,K) MUT is given as [2,3,4,13]

(1) $$h_{k}=\Psi_{k}^{\left(0\right)}a\left(\theta_{k}^{\left(0\right)}\right)+\sum_{l=1}^{N_{p}}\Psi_{k}^{\left(l\right)}a\left(\theta_{k}^{\left(l\right)}\right),$$

where $\Psi_{k}^{\left(0\right)}$ represents the complex gain and $a\left(\theta_{k}^{\left(0\right)}\right)$ represents the array steering vector for the LOS path, $\Psi_{k}^{\left(l\right)}$ are the complex gains and $a\left(\theta_{k}^{\left(l\right)}\right)$ are the steering vectors $\left(l=1,2,\ldots ,N_{p}\right)$ for the non-line-of-sight (NLOS) paths that exist between the gNB and the *k*th MUT, and $N_{p}$ represents the number of NLOS propagation paths [2,3,4].

For a conventional uniform linear array with *N* antennas, the array steering vector is given as [2,3,4,9,13,16]

(2) $$a\left(\theta \right)=\frac{1}{\sqrt{N}}{\left[e^{-j2\pi \theta m}\right]}_{m\in \ell \left(N\right)},$$

where $\ell \left(N\right)=\left\{n-\left(N-1\right)/2,n=0,1,2,\ldots ,N-1\right\}$ is a set of indices that are symmetric and centered around zero (i.e., the reference element) [9]. The spatial direction is given by θ=(dsinϕ)/λ, where ϕ is the physical direction of the corresponding path, such that $-\frac{\pi }{2}\leq \phi \leq \frac{\pi }{2}$, λ is the wavelength of the signal, and d=λ/2 is the distance between antenna elements [2,3,4].

The conventional mmWave channels in the spatial domain can be converted to beam spatial channels by employing a lens antenna array [2,4]. As shown in Figure 1b, the gNB employs a lens antenna array, which can be represented mathematically by an N×N discrete Fourier transform matrix U that contains the array steering vectors [2,4]. The matrix U can be expressed as [2,4]

(3) $$U={\left[a\left({\tilde{\theta }}_{1}\right),a\left({\tilde{\theta }}_{2}\right),\ldots ,a\left({\tilde{\theta }}_{N}\right)\right]}^{H},$$

where ${\tilde{\theta }}_{n}=\frac{1}{N}\left(n-\frac{N+1}{2}\right)$ for n=1,2,…,N denotes the predefined spatial directions [2,4]. Therefore, the beamspace channel matrix $\bar{H}$ for serving *K* MUTs is given as [2,4]

(4) $$\bar{H}=\left[{\mathrm{Uh}}_{1},{\mathrm{Uh}}_{2},\ldots ,{\mathrm{Uh}}_{K}\right]=\left[{\bar{h}}_{1},{\bar{h}}_{2},\ldots ,{\bar{h}}_{K}\right],$$

where $h_{k}$ and ${\bar{h}}_{k}$ are the spatial and beamspace channel vectors between the gNB and the *k*th MUT, respectively [2,4]. Using the spatial direction of the channel θ, the steering vectors of the LOS path and the NLOS paths for the *k*th MUT can be obtained from (2).

The number of NLOS paths $N_{p}$ is typically considerably less than the number of gNB antennas *N* because there are a limited number of dominating scatterers in the mmWave channel [2,8]. Consequently, each beamspace channel vector has a substantially smaller number of dominant elements than its dimension [2,4]. Because of this sparse nature of the mmWave channel, beamspace mMIMO NOMA undergoes a beam selection process to select the dominant beams in $\bar{H}$, which reduces the number of RF chains [2,4]. Many beam selection algorithms have been proposed, for example, maximal-magnitude-based beam selection [8], greedy beam selection [10], interference aware (IA) beam selection technique [11], and the maximization of the SINR [9] selection criteria, to serve all MUTs without notable loss in performance [2]. In this work, we consider the maximal-magnitude-beam selection technique, where each MUT selects the beam with the largest magnitude [2,11]. Specifically, the elements of the beamspace channel ${\bar{h}}_{k}$ are arranged in descending order of magnitude [11]. Moreover, the beam corresponding to the channel coefficient with the largest magnitude is selected for each MUT [11]. Therefore, following the beam selection, the signal vector received by the MUTs can be expressed as [2,4]

(5) $$y={\bar{H}}_{r}^{H}W_{r}Px+n,$$

where $n∼\mathrm{CN}(0,\sigma^{2}I_{K})$ is the K×1 additive white Gaussian noise (AWGN), x is the K×1 vector whose elements are the transmitted signal for all *K* MUTs with normalized power, such that $E\left({\mathrm{xx}}^{H}\right)=I_{K}$, $P=\mathrm{diag}[\sqrt{p_{1}},\sqrt{p_{2}},\ldots ,\sqrt{p_{K}}]$ is the transmit power matrix for *K* MUTs satisfying $\sum_{k=1}^{K}p_{k}\leq P$, such that *P* is the maximum power transmitted by the gNB, $W_{r}$ is the dimension-reduced digital precoding matrix whose row order is equal to $\left|B\right|=N_{RF}<N$, ${\bar{H}}_{r}=\bar{H}{\left(i,:\right)}_{i\in B}$ of size |B|×K is the dimension-reduced beamspace channel matrix, and B is the set of selected beam indices [2]. As a result of beam selection, the number of RF blocks in beamspace MIMO systems can be reduced, thus reducing the energy usage and hardware complexity in mmWave mMIMO systems [2].

**Figure 2:**
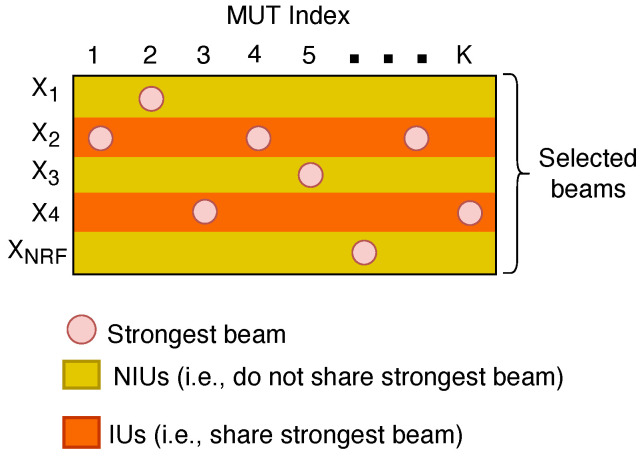
Illustration of the IUs and NIUs produced by beam selection.

Furthermore, there is a high probability that some MUTs will have the same beam index as their strongest beam [11]. As a result, the MUTs can be segregated into non-interfering users (NIUs) and interfering users (IUs), where NIUs are MUTs that do not share the same strongest beam and IUs are MUTs that share the same strongest beam [11], as illustrated in Figure 2. Let $X_{m}$ represent the set of indices of MUTs served by the *m*th beam for $m=1,2,\ldots ,N_{RF}$, such that $X_{i}\cap X_{j}=⌀$ for i≠j, $\sum_{m=1}^{N_{RF}}\left|X_{m}\right|=K$ [4]. If set $X_{m}$ has only one element, it is referred to as the set of NIUs. On the other hand, if set $X_{m}$ has more than one element, it becomes the set of IUs [11]. From Figure 2, the set consisting of the sets of NIUs is $S_{NIUs}=\left\{X_{1},X_{3},X_{N_{RF}}\right\}$ and the set consisting of the sets of IUs is $S_{IUs}=\left\{X_{2},X_{4}\right\}$. Consequently, the subset of set $S_{IUs}$ is referred to as a NOMA group or NOMA MUTs [2]; both terms are used interchangeably in this paper. Moreover, the NOMA MUTs are arranged in decreasing order of their channel quality $∥{\bar{h}}_{m,i}∥$, i.e., $∥{\bar{h}}_{m,1}{∥}^{2}\geq ∥{\bar{h}}_{m,2}{∥}^{2}\geq \ldots \geq {∥{\bar{h}}_{m,|X_{m}|}∥}^{2}$, where ${\bar{h}}_{m,i}$ is the $N_{RF}\times 1$ beamspace channel vector between the gNB and the *i*th MUT in the *m*th beam after beam selection [2]. Therefore, the MUT whose equivalent channel magnitude is $∥{\bar{h}}_{m,|X_{m}|}{∥}^{2}$ is regarded as the cell-edge MUT, whereas the MUT whose equivalent channel magnitude is $∥{\bar{h}}_{m,1}{∥}^{2}$ is assumed to be the MUT closest to the gNB.

**Figure 3:**
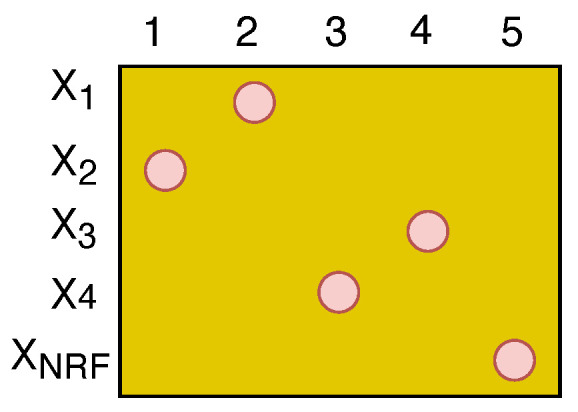
Illustration of the equivalent channel matrix $\tilde{H}$ of size $N_{RF}\times N_{RF}.$

To design the digital precoding matrix, the dimension-reduced beamspace channel matrix ${\bar{H}}_{r}$ can be further reduced to the equivalent channel matrix $\tilde{H}$ of size $N_{RF}\times N_{RF}$ by considering all columns in ${\bar{H}}_{r}$ corresponding to the elements in set $S_{NIUs}$ and the column corresponding to the maximal element within subsets $X_{2}$ and $X_{4}$ of set $S_{IUs}$ [3]. Consequently, the resulting channel matrix becomes the equivalent channel matrix $\tilde{H}$ of size $N_{RF}\times N_{RF}$, as illustrated in Figure 3. The digital precoding matrix $\tilde{W}$ of size $N_{RF}\times N_{RF}$ based on ZF is given as [4]

(6) $$\tilde{W}=\tilde{H}{\left({\tilde{H}}^{H}\tilde{H}\right)}^{-1}.$$

The digital precoding vectors should be normalized to prevent the recurrence of power allocation calculations because all MUTs sharing the same beam are given the same precoding vector but different power levels [2]. Thus, the precoding vector for the *m*th ($m=1,2,\cdots ,N_{RF}$) beam is [3,4]

(7) $$w_{m}=\frac{{\tilde{w}}_{m}}{∥{\tilde{w}}_{m}{∥}_{2}},$$

where ${\tilde{w}}_{m}=\tilde{W}\left(:,m\right)$ is the $N_{RF}\times 1$ precoding vector of the *m*th beam before normalization [2]. With this precoding, the MUT closest to the gNB in each beam can completely eliminate the inter-beam interferences [4].

For the NOMA MUTs, the *i*th MUT in the *m*th beam can successively detect and remove the signal of the *n*th MUT from the received signal using SIC (The knowledge of the channel state information (CSI) at both the gNB and the MUTs is crucial for capacity-approaching performance [46]. Moreover, due to the sparsity of the beamspace channel, compressed sensing or dictionary learning-based techniques can be utilized to estimate the channel with highly reduced pilot overhead reliably [46,47]. As a result, we assume the CSI is known at both the gNB and the MUTs.) for $i<n\leq |X_{m}|$ [4]. Subsequently, the *i*th MUT decodes its own signal [4]. Therefore, the signal $y_{m,i}$ received by the *i*th MUT in the *m*th beam is given as [2,3,4]

(8) $$\begin{array}{cc} y_{m,i} & =\underset{\mathrm{desired}\;\mathrm{signal}}{\underset{︸}{{\bar{h}}_{m,i}^{H}w_{m}\sqrt{p_{m,i}}x_{m,i}}}\\ & +\underset{\mathrm{intra}-\mathrm{beam}\;\mathrm{interference}}{\underset{︸}{{\bar{h}}_{m,i}^{H}w_{m}\sum_{s=1}^{i-1}\sqrt{p_{m,s}}x_{m,s}+{\bar{h}}_{m,i}^{H}w_{m}\sum_{s=i+1}^{|X_{m}|}\sqrt{p_{m,s}}x_{m,s}}}\;\\ & +\underset{\mathrm{inter}-\mathrm{beam}}{\underset{︸}{{\bar{h}}_{m,i}^{H}\sum_{t\neq m}\sum_{s=1}^{|X_{t}|}w_{t}\sqrt{p_{t,s}}x_{t,s}}}\;\mathrm{interference}+\underset{\mathrm{noise}}{\underset{︸}{n_{m,i}}}\;, \end{array}$$

where $p_{m,i}$ and $x_{m,i}$ are the transmit power and the signal sent to the *i*th MUT in the *m*th beam, respectively, and $n_{m,i}∼\mathrm{CN}\left(0,\sigma^{2}\right)$ is additive Gaussian noise [2,3,4].

From (8), the SINR at the *i*th MUT in the *m*th beam for decoding its signal can be expressed as [2,3,4]

(9) $$\gamma_{m,i}=\frac{|{\bar{h}}_{m,i}^{H}\left(\sqrt{p_{m,i}}w_{m}\right){|}^{2}}{\zeta_{m,i}},$$

where

(10) $$\begin{array}{c} \zeta_{m,i}=\sum_{s=1}^{i-1}|{\bar{h}}_{m,i}^{H}\left(\sqrt{p_{m,s}}w_{m}\right){|}^{2}+\sum_{t\neq m}\sum_{s=1}^{|X_{t}|}{\left|{\bar{h}}_{m,i}^{H}\left(\sqrt{p_{t,s}}w_{t}\right)\right|}^{2}+\sigma^{2}. \end{array}$$

Therefore, the feasible rate of the *i*th MUT in the *m*th beam is [2,3,4]

(11) $$R_{m,i}=\log_{2}\left(1+\gamma_{m,i}\right).$$

To ensure successful SIC, each MUT with an index smaller than the *i*th MUT in the *m*th beam should be able to detect the signal of the *i*th MUT [2]. Thus, the feasible sum rate of the beamspace mMIMO NOMA scheme is [2,3,4]

(12) $$R_{sum}=\sum_{m=1}^{N_{RF}}\sum_{i=1}^{|X_{m}|}R_{m,i}.$$

The feasible sum rate in (12) can be maximized by using the precoding vectors obtained from (6) and performing power allocation optimization to minimize the intra-beam interferences [4]. Therefore, a good power allocation strategy is essential for increasing the spectral efficiency in beamspace mMIMO NOMA systems [4]. For the dynamic power allocation in [4], the power allocation optimization considered both inter- and intra-group power optimizations. Hence, the optimal power allocation is achieved by minimizing the inter- and intra-group interferences on one hand while increasing the feasible sum rate on the other hand [4]. As such, we employed the iterative dynamic power allocation strategy proposed in [4], which is dependent on the minimum mean square error (MMSE) detection problem.

Since MMSE detection is utilized at the MUT to obtain $x_{m,i}$ from $y_{m,i}$ (8), the mean square error (MSE) problem is derived as [4]

(13) $$c_{m,i}^{opt}=\arg\min_{c_{m,i}}\;e_{m,i},$$

where

(14) $$e_{m,i}=E[|x_{m,i}-c_{m,i}y_{m,i}{|}^{2}].$$

Therefore,

(15) $$\begin{array}{cc} e_{m,i} & =|1-c_{m,i}\sqrt{p_{m,i}}{\bar{h}}_{m,i}^{H}w_{m}{|}^{2}+|c_{m,i}{|}^{2}{\left|{\bar{h}}_{m,i}^{H}w_{m}\right|}^{2}\sum_{s=1}^{i-1}p_{m,s}\\ & +|c_{m,i}{|}^{2}\sum_{t\neq m}^{N_{RF}}|{\bar{h}}_{m,i}^{H}w_{t}{|}^{2}\sum_{s=1}^{|X_{t}|}p_{t,s}+{\left|c_{m,i}\right|}^{2}\sigma^{2}, \end{array}$$

where $c_{m,i}$ is the channel equalization coefficient (CEC), and the optimum CEC, denoted by $c_{m,i}^{opt}$, that minimizes the MSE can be determined by solving $\frac{\partial e_{m,i}}{\partial c_{m,i}}{|}_{c_{m,i}^{opt}}=0$ [4]. Thus,

(16) $$-\sqrt{p_{m,i}}w_{m}^{H}{\bar{h}}_{m,i}+c_{m,i}^{opt}\left(p_{m,i}{\left|{\bar{h}}_{m,i}^{H}w_{m}\right|}^{2}+\zeta_{m,i}\right)=0.$$

Therefore,

(17) $$c_{m,i}^{opt}=\frac{\sqrt{p_{m,i}}w_{m}^{H}{\bar{h}}_{m,i}}{p_{m,i}{\left|{\bar{h}}_{m,i}^{H}w_{m}\right|}^{2}+\zeta_{m,i}}.$$

Thus, the MMSE $e_{m,i}^{opt}$ is obtained by substituting (17) into (14) [4]:

(18) $$\begin{array}{cc} e_{m,i}^{opt} & =1-\frac{2p_{m,i}{\left|{\bar{h}}_{m,i}^{H}w_{m}\right|}^{2}}{p_{m,i}{\left|{\bar{h}}_{m,i}^{H}w_{m}\right|}^{2}+\zeta_{m,i}}+\frac{p_{m,i}{\left|{\bar{h}}_{m,i}^{H}w_{m}\right|}^{2}}{p_{m,i}{\left|{\bar{h}}_{m,i}^{H}w_{m}\right|}^{2}+\zeta_{m,i}}\\ & =1-\frac{p_{m,i}{\left|{\bar{h}}_{m,i}^{H}w_{m}\right|}^{2}}{p_{m,i}{\left|{\bar{h}}_{m,i}^{H}w_{m}\right|}^{2}+\zeta_{m,i}}\;. \end{array}$$

It was proved in [4] that

(19) $$R_{m,i}=\log_{2}\left(1+\gamma_{m,i}\right)=\max_{c_{m,i}}\left(-\log_{2}e_{m,i}\right).$$

Proposition 1 in [4] verified that given a function $f\left(a\right)=-\frac{ab}{\ln2}+\log_{2}a+\frac{1}{\ln2}$, we will have

(20) $$\max_{a>0}\;f\left(a\right)=-\log_{2}b,$$

where $a^{opt}=\frac{1}{b}$ is the optimal value of *a* [4]. Then, $a_{m,i}^{opt}$ can be obtained as

(21) $$a_{m,i}^{opt}=\frac{1}{e_{m,i}^{opt}}.$$

After obtaining the optimal $e_{m,i}^{opt}$ and $a_{m,i}^{opt}$ from (18) and  (21), the optimal power $p_{m,i}^{opt}$ is given as [4]

(22) pm,iopt=am,ioptRecm,iopth¯m,iHwmτ2,

where

(23) $$\begin{array}{cc} \tau & =\sum_{s=i}^{|X_{m}|}a_{m,s}^{opt}|c_{m,s}^{opt}{|}^{2}∥{\bar{h}}_{m,s}^{H}w_{m}{∥}^{2}+\sum_{v\neq m}\sum_{s=1}^{|X_{v}|}a_{v,s}^{opt}|c_{v,s}^{opt}{|}^{2}{∥{\bar{h}}_{v,s}^{H}w_{m}∥}^{2}\\ & +\lambda -\mu_{m,i}∥{\bar{h}}_{m,i}^{H}w_{m}{∥}^{2}+\sum_{s=i+1}^{|X_{m}|}\mu_{m,s}\eta ∥{\bar{h}}_{m,s}^{H}w_{m}{∥}^{2}+\sum_{v\neq m}\sum_{s=1}^{|X_{v}|}\mu_{v,s}\eta {∥{\bar{h}}_{v,s}^{H}w_{m}∥}^{2}, \end{array}$$

$\eta =2^{Rmin}-1$, where $R_{min}$ is the minimum guaranteed data rate for each MUT, λ≥0, and $\mu_{m,i}\geq 0$, such that $m=1,2,\cdots ,N_{RF}$ and $i=1,2,\cdots ,|X_{m}|$ [4].

At the *t*th iteration, the optimal $c_{m,i}^{opt(t)}$, $a_{m,i}^{opt(t)}$, and $p_{m,i}^{opt(t)}$ can be calculated from (17), (21), and (22), respectively [4]. Each iteration produces optimal solutions of $c_{m,i}^{opt(t)}$, $a_{m,i}^{opt(t)}$, and $p_{m,i}^{opt(t)}$ [4]. As a result, at each iteration, these optimal values are updated and either increase or retain the value of the feasible sum rate $R_{sum}$ [4]. For more details on the iterative power allocation procedure, refer to [4].

## 3. Proposed Threshold-Based Cooperative Relaying

### 3.1. Spectral Efficiency

As mentioned in the previous section, the MUTs that have selected the same beam form a NOMA group [2,4]. As a result, the threshold-based user-assisted cooperative relaying in beamspace mMIMO NOMA is implemented within the NOMA group [3]. In this technique, MUTs are allocated the optimized power as described in the previous section. The sum of these allocated powers for all MUTs within the NOMA cluster is the total transmit power $p_{m}$ for that beam [3]. The symbols from the gNB are forwarded to the destination (cell-edge MUT) by employing the intermediate MUTs within the NOMA group as relay stations. In this work, we assume that the channel remains constant throughout the entire symbol transmission (i.e., a slow fading channel). Even though each intermediate MUT can detect and remove the signal of the cell-edge MUT from its received signal using SIC and decode its own signal, an intermediate MUT can participate in relaying the symbols to the edge-cell MUT only if its SINR for decoding the signal of the cell-edge MUT satisfies the predetermined threshold requirement $\left(\gamma_{th}\right)$ and updates the gNB about its SINR status through the feedback channel. Unlike in [33,39,44], if no intermediate MUT satisfies the threshold, the cell-edge MUT will utilize the signal received from the gNB. In [40,41], the messages for the relay and destination are split into common and private messages. The relay participates in relaying only if its private message is unavailable, which is regarded as partial cooperative relaying. Contrary to [40,41], the gNB performs superposition coding to encode the symbols of the intermediate MUTs and the cell-edge MUT and transmits the composite signal to the intermediate MUTs and the cell-edge MUT in phase 1. The relaying MUTs decode and retransmit the symbols of the cell-edge MUT in phase 2 (i.e., half-duplex decode-and-forward relaying). To improve the data reliability and maximize the feasible sum rate, the cell-edge MUT performs maximal ratio combining (MRC) to combine these symbols received from the intermediate relaying MUTs in phase 2 assuming perfect knowledge of the channel gains. Therefore, the cell-edge MUT receives symbols from the gNB in phase 1 and from the MRC path (i.e., relaying MUTs) in phase 2 and utilizes both to maximize SINR and error correction. A detailed description of the user-assisted relay selection strategy is given as Algorithm 1.
**Algorithm 1:** Threshold-based user-assisted relay selection**Input: **$h_{gNB,i}$, $h_{gNB,|X_{m}|}$, $p_{m,i}$, $\gamma_{th}$, R={1,⋯,u}**Output: **$D_{m}$ Initialization: $D_{m}=⌀$ No relaying MUT is selected. **for** i=1 to *u* **do**  **if** $\gamma_{gNB,i}\geq \gamma_{th}$ **then**   $D_{m}\leftarrow D_{m}\cup \left\{i\right\}$  **end if** **end for** Return $D_{m}$

Let R={1,⋯,u}$\left(u=|X_{m}|-1\right)$ be the set of indices of intermediate MUTs in the *m*th beam. As a result, the intermediate MUTs in set *R* that meet the SINR threshold $\left(\gamma_{th}\right)$ are designated as relaying MUTs. In the *t*th time slot, the gNB transmits the superimposed signal to all the relays in set *R* and the cell-edge MUT, which can be expressed as

(24) $$x_{m}=\sqrt{a_{1}p_{m}}x_{m,1}+\cdots +\sqrt{a_{u}p_{m}}x_{m,u}+\sqrt{a_{|X_{m}|}p_{m}}x_{m,|X_{m}|},$$

where $x_{m,i}$ represents the symbol of the *i*th MUT in the *m*th beam with normalized power, such that $E[|x_{m,i}{|}^{2}]=1$, $p_{m}$ is the total optimized transmit power allocated for the MUTs in the *m*th beam, and $a_{i}$ is the power allocation coefficient for each MUT, such that $a_{1}+\cdots +a_{u}+a_{|X_{m}|}=1$ and $a_{|X_{m}|}>a_{u}>\cdots >a_{1}$ [3,35]. The relays successively decode the symbols intended for the cell-edge MUT and forward them in the second time slot. The cell-edge MUT receives and combines the symbols sent from all the relaying MUTs using MRC to maximize the SINR. The signals received by the relaying MUTs and the cell-edge MUT in the *t*th time slot are given as

(25) $$y_{m,i}=h_{gNB,i}x_{m}+n_{m,i},$$

(26) $$y_{m,|X_{m}|}=h_{gNB,|X_{m}|}x_{m}+n_{m,|X_{m}|},$$

where $h_{gNB,i}={\bar{h}}_{m,i}^{H}w_{m}$ and $h_{gNB,|X_{m}|}={\bar{h}}_{m,|X_{m}|}^{H}w_{m}$, $i\in D_{m}$, are the effective mmWave beamspace channels between the gNB and the *i*th relaying MUT and between the gNB and the cell-edge MUT, respectively. Moreover, $x_{m}$ is the superimposed signal transmitted by the gNB, and $n_{m,i}$ and $n_{m,|X_{m}|}$ are the AWGNs at the relaying MUTs and cell-edge MUT with zero mean and variance $\sigma^{2}$, respectively. In the *t*th time slot, the cell-edge MUT decodes its own symbols while treating the symbols intended for other MUTs as noise. Moreover, the inter-beam interferences are eliminated using the beam-based ZF precoding introduced in Section 2. Therefore, the received SINR at the cell-edge MUT acquiring its symbols is given as

(27) $$\gamma_{gNB,|X_{m}|}^{m}=\frac{|h_{gNB,|X_{m}|}{|}^{2}a_{|X_{m}|}p_{m}}{\sum_{i=1}^{u}{\left|h_{gNB,i}\right|}^{2}a_{i}p_{m}+\sigma^{2}},$$

and the received SINR at the *i*th relaying MUT decoding the symbols of the cell-edge MUT in the *t*th time slot is given by

(28) $$\gamma_{gNB,i}^{m}=\frac{|h_{gNB,i}{|}^{2}a_{|X_{m}|}p_{m}}{\sum_{j=1}^{u}{\left|h_{gNB,i}\right|}^{2}a_{j}p_{m}+\sigma^{2}}.$$

Assuming successful SIC, the received SINR at the *i*th relaying MUT decoding its own symbols is

(29) $$\gamma_{gNB,i}^{m(t)}=\frac{|h_{gNB,i}{|}^{2}a_{i}p_{m}}{\sum_{x=1}^{x<i}{\left|h_{gNB,x}\right|}^{2}a_{x}p_{m}+\sigma^{2}}.$$

In the $\left(t+1\right)$th time slot, the cooperating relays retransmit the symbols intended for the cell-edge MUT. Assuming perfect decoding of the symbols by the cooperating relays in the *t*th time slot, the received signal at the cell-edge MUT in the $\left(t+1\right)$th time slot from all the relaying MUTs is expressed as

(30) $$y_{i,|X_{m}|}^{m}=\sum_{i\in D_{m}}h_{i,|X_{m}|}\sqrt{b_{i}p_{m}}x_{m,|X_{m}|}+n_{m,|X_{m}|},$$

where $h_{i,|X_{m}|}$ is the channel coefficient between the *i*th relay and the cell-edge MUT, $b_{i}$ is the power allocation coefficient at the *i*th relay, such that $\sum_{i\in D_{m}}b_{i}=1$ and $b_{i}>b_{j}$ for i>j, and $n_{m,|X_{m}|}$ is the AWGN at the cell-edge MUT with zero mean and variance $\sigma^{2}$ [3,35]. Then, the cell-edge MUT decodes the strongest signal and performs SIC to decode the remaining signals successively. Therefore, the cell-edge MUT decodes the strongest signal while treating the remaining signals as noise. The received SINR at the cell-edge MUT for decoding signal $x_{|X_{m}|}$ from the *i*th relay in (30) is expressed as

(31) $$\gamma_{i,|X_{m}|}^{m}=\frac{|h_{i,|X_{m}|}{|}^{2}b_{i}p_{m}}{\sum_{j\in D_{m}\setminus \left\{i\right\}}{\left|h_{j,|X_{m}|}\right|}^{2}b_{j}p_{m}+\sigma^{2}}.$$

Then, the cell-edge MUT implements MRC for the signals received from the relaying paths in the (t+1)th time slot to maximize the SINR, and the effective SINR is expressed as

(32) $$\gamma_{mrc}^{m}=\sum_{i=1}^{u}\gamma_{i,|X_{m}|}^{m}.$$

For the successful decoding of the symbols at the relays and the cell-edge MUT, the rates of these symbols must be lower than the rate given by the Shannon formula [35]. Utilizing NOMA, two orthogonal time slots are sufficient to perform the cooperative transmission [48]. Therefore, the message transmitted in phase 1 must be coded with a rate of $2R_{min}$ to achieve an average end-to-end rate of $R_{min}$. As a result of (27), (31) and (32), the feasible rate $C_{|X_{m}|}$ at the cell-edge MUT decoding symbol $x_{|X_{m}|}$ is given as

(33) $$\begin{array}{c} C_{|X_{m}|}^{m}=\frac{1}{2}\min\left\{\log_{2}\left(1+\gamma_{gNB,|X_{m}|}^{m}\right),\log_{2}\left(1+\gamma_{mrc}^{m}\right)\right\}. \end{array}$$

The feasible sum rate at the cell-edge MUTs in all the CRS beams (CRS beams refer to the NOMA groups, where user-assisted cooperative relaying is implemented.) is given as

(34) $$C_{sum}=\sum_{x=1}^{M\leq N_{RF}}C_{|X_{m}|,x}^{m},$$

where *M* is the number of beams selected by more than one MUT. Therefore, the total feasible sum rate of the system is given as

(35) $$C^{Total}=\sum_{m=1}^{N_{RF}}\sum_{i=1}^{|X_{m}|-1}R_{m,i}+C_{sum}.$$

### 3.2. Outage Probability

In this section, an analytic closed-form expression is derived for the outage probability at the cell-edge MUT in the threshold-based user-assisted cooperative relaying in the beamspace mMIMO NOMA scheme. As indicated in Section 2, the minimum data rate required for successful transmission for each MUT is $R_{min}$. An outage event at the cell-edge MUT occurs when the capacity $C_{i,|X_{m}|}$ of the link between the cell-edge MUT and the *i*th relaying MUT is less than $R_{min}$ (i.e., $C_{i,|X_{m}|}<R_{min}\forall i$) and when the capacity $C_{gNB,|X_{m}|}$ of the link between the gNB and the cell-edge MUT is less than $R_{min}$ (i.e., $C_{gNB,|X_{m}|}<R_{min}$ ). Hence, the outage probability along the gNB and cell-edge MUT link is given as

(36) $$\begin{array}{cc} P_{gNB,|X_{m}|}^{m} & =\mathrm{Pr}\left(\log_{2}\left(1+\gamma_{gNB,|X_{m}|}^{m}\right)<R_{min}\right)\\ \; & =\mathrm{Pr}\left(\gamma_{gNB,|X_{m}|}^{m}<2^{R_{min}}-1\right)=1-e^{\left(-\frac{2^{R_{min}}-1}{\gamma_{gNB,|X_{m}|}^{m}}\right)}. \end{array}$$

An outage along the MRC path will occur only if the SINRs of all the links between the relaying MUTs and cell-edge MUT is less than the minimum rate. Therefore, the outage along the MRC path is given as

(37) $$\begin{array}{cc} P_{\gamma_{mrc}}^{m} & =\mathrm{Pr}\left(\frac{1}{2}\min\left\{\log_{2}\left(1+\gamma_{gNB,i}^{m}\right),\log_{2}\left(1+\gamma_{mrc}^{m}\right)\right\}<R_{min}\right)\\ \; & =\mathrm{Pr}\left(\frac{1}{2}\log_{2}\left(1+\gamma_{gNB,i}^{m}\right)<R_{min}\right)+\mathrm{Pr}\left(\frac{1}{2}\log_{2}\left(1+\gamma_{gNB,i}^{m}\right)\geq R_{min}\right)\\ \; & \times \mathrm{Pr}\left(\frac{1}{2}\log_{2}\left(1+\gamma_{mrc}^{m}\right)<R_{min}\right)\\ \; & =\mathrm{Pr}\left(\gamma_{gNB,i}^{m}<2^{2R_{min}}-1\right)+\mathrm{Pr}\left(\gamma_{gNB,i}^{m}\geq 2^{2R_{min}}-1\right)\times \mathrm{Pr}\left(\gamma_{mrc}^{m}<2^{2R_{min}}-1\right)\\ \; & =1-e^{\left(-\frac{2^{2R_{min}}-1}{\gamma_{gNB,i}^{m}}\right)}+e^{\left(-\frac{2^{2R_{min}}-1}{\gamma_{gNB,i}^{m}}\right)}\left[1-e^{\left(-\frac{2^{2R_{min}}-1}{\gamma_{mrc}^{m}}\right)}\right]\\ \; & =1-e^{\left(-\frac{2^{2R_{min}}-1}{\gamma_{gNB,i}^{m}}\right)}e^{\left(-\frac{2^{2R_{min}}-1}{\gamma_{mrc}^{m}}\right)}. \end{array}$$

Since the outages on both links are mutually independent, the closed-form expression for the outage probability at the cell-edge MUT in the *m*th beam is given as

(38) $$\begin{array}{cc} P_{out}^{m} & =\left[1-e^{\left(-\frac{\alpha }{\gamma_{gNB,|X_{m}|}^{m}}\right)}\right]\times \left[1-e^{\left(-\frac{\beta }{\gamma_{gNB,i}^{m}}\right)}e^{\left(-\frac{\beta }{\gamma_{mrc}^{m}}\right)}\right], \end{array}$$

where $\beta =2^{2R_{min}}-1$ and $\alpha =2^{R_{min}}-1$.

At a high SNR (i.e., when $\gamma_{gNB,|X_{m}|}^{m}$, $\gamma_{gNB,i}^{m}$, $\gamma_{mrc}^{m}\gg 0$), we can obtain an approximation of the outage probability using first-order Taylor approximation (where $e^{-x}\approx 1-x$ for sufficiently small x) [48]. Consequently, the approximation of the outage probability is expressed as

(39) $$P_{out}^{m}\approx \frac{\alpha \beta }{\gamma_{gNB,|X_{m}|}^{m}\gamma_{gNB,i}^{m}}\left(1+\frac{\gamma_{gNB,i}^{m}}{\gamma_{mrc}^{m}}\right).$$

## 4. Simulation Results

This section provides simulation results that validate the performance of the proposed threshold-based user-assisted CRS beamspace mMIMO NOMA system. We considered a downlink mmWave mMIMO system where the gNB is equipped with N=64 antennas and communicates with *K* MUTs simultaneously [2]. The total transmit power is $P_{t}=32$ mW (15 dBm) [4] and the minimum guaranteed target rate for each MUT is considered to be $R_{\min}=0.5$ bps/Hz [34]. The SNR is expressed as SNR $=1/\sigma^{2}$ [48]. The channels between the gNB and each MUT are assumed to have one LOS component and $N_{p}=2$ NLOS components [4]. According to some studies, 1) the channel parameters of the *k*th MUT are $\;\beta_{k}^{\left(0\right)}∼\mathrm{CN}\left(0,1\right),\;\beta_{k}^{\left(l\right)}∼\mathrm{CN}\left(0,10^{-1}\right)$ for $1\leq l\leq N_{p},\;2)\;\theta_{k}^{\left(0\right)}$ and $\theta_{k}^{\left(l\right)}$ are random variables uniformly distributed within $\left[-\frac{1}{2},\frac{1}{2}\right]$ for $1\leq l\leq N_{p}$ [3,4]. The means of the respective channel gains between the gNB and the *i*th relay and between the gNB and the cell-edge MUT are obtained to determine the mmWave channel gains between the relays and the cell-edge MUT in the *m*th beam, which are designated as $h_{i,|X_{m}|}$, such that $i\in D_{m}$ [3]. Four baseline systems are taken into account in this simulation for comparison:

- A CRS beamspace mMIMO NOMA system, which integrates a beamspace mMIMO system with NOMA and a multi-hop CRS [3].
- A beamspace MIMO-NOMA system, which integrates NOMA and a beamspace MIMO system to serve $K\geq N_{RF}$ [4] MUTs.
- A MIMO-OMA system [49] with $N_{RF}\leq K$, in which OMA is performed for IUs within the same beam, and orthogonal frequency resources are allocated for MUTs within the beam.
- The proposed threshold-based user-assisted CRS beamspace mMIMO NOMA system, which integrates beamspace mMIMO NOMA and a threshold-based user-assisted CRS.

Dominant scatterers are limited in mmWave communications, and most of the beam energy is concentrated in the LOS component [4,8,13]. Moreover, the unavailability of the LOS component results in poor channel correlation in the same beam [4]. As such, we assumed that a direct path exists between the gNB and the cell-edge MUT and between the gNB and the user-assisted relays. The efficiency of the four baseline systems mentioned above is assessed in terms of outage probability, energy efficiency, and spectral efficiency when the delay-intolerant cell-edge MUT is constrained to receive all symbols within a time frame that is arbitrarily chosen to be T=4 transmission slots. Otherwise, the delay-intolerant cell-edge MUT will discard the remaining symbols, resulting in an error.

### 4.1. Spectral Efficiency

**Figure 4:**
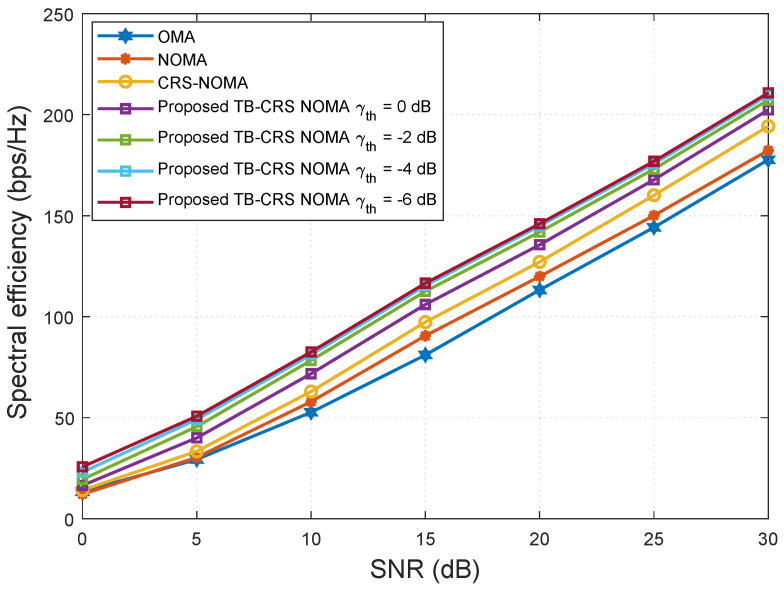
Spectral efficiency versus SNR for K = 32 MUTs.

Figure 4 shows the spectral efficiency versus SNR when the number of MUTs served by the gNB is K=32. The proposed threshold-based user-assisted CRS beamspace mMIMO NOMA scheme is assessed for various relay selection SINR threshold values $\gamma_{th}=\left\{0,-2,-4,-6\right\}$ dB [50] and then compared with the baseline systems. The proposed threshold-based user-assisted CRS beamspace mMIMO NOMA method achieves higher spectral efficiency than all of the baseline systems, and the performance gap remains constant with increasing SNR, as shown in Figure 4. When the SINR threshold value is switched from $\gamma_{th}=0$ to −2 dB, the proposed system achieves increased spectral efficiency. However, when $\gamma_{th}=-4$ dB, it shows negligible performance gain compared with that when $\gamma_{th}=-6$ dB. Therefore, further reduction in the SINR threshold value beyond $\gamma_{th}=-4$ dB will not result in a large spectral efficiency gain.

**Figure 5:**
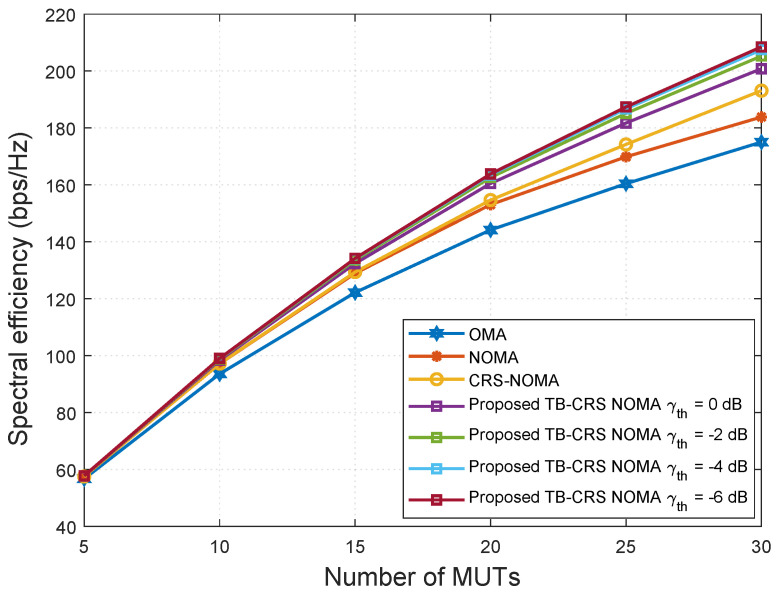
Spectral efficiency versus the number of MUTs when SNR = 20 dB.

Figure 5 shows the spectral efficiency versus the number of MUTs when SNR is 20 dB. The proposed threshold-based user-assisted CRS beamspace mMIMO NOMA scheme is analysed for various relay selection SINR threshold values $\gamma_{th}=\left\{0,-2,-4,-6\right\}$ dB [50] and then compared with the baseline systems. The proposed threshold-based user-assisted CRS beamspace mMIMO NOMA method achieves higher spectral efficiency than all of the baseline systems, and the performance gap increases as the number of MUTs increases, as shown in Figure 5. This is because, even with the highest SINR threshold value, $\gamma_{th}=0$ dB, the cell-edge MUT can still receive signals from the gNB when no intermediate MUT meets the SINR threshold requirement. When the SINR threshold value is switch from $\gamma_{th}=0$ to −2 dB, the performance gap between the proposed scheme and the baseline systems increases further. This is because, when the SINR threshold value is decreased, a greater number of intermediate MUTs can become relay MUTs. However, when $\gamma_{th}=-4$ dB, there is negligible performance gain compared with that when $\gamma_{th}=-6$ dB. Therefore, further reduction in the SINR threshold value beyond $\gamma_{th}=-4$ dB results in negligible spectral efficiency gain.

**Figure 6:**
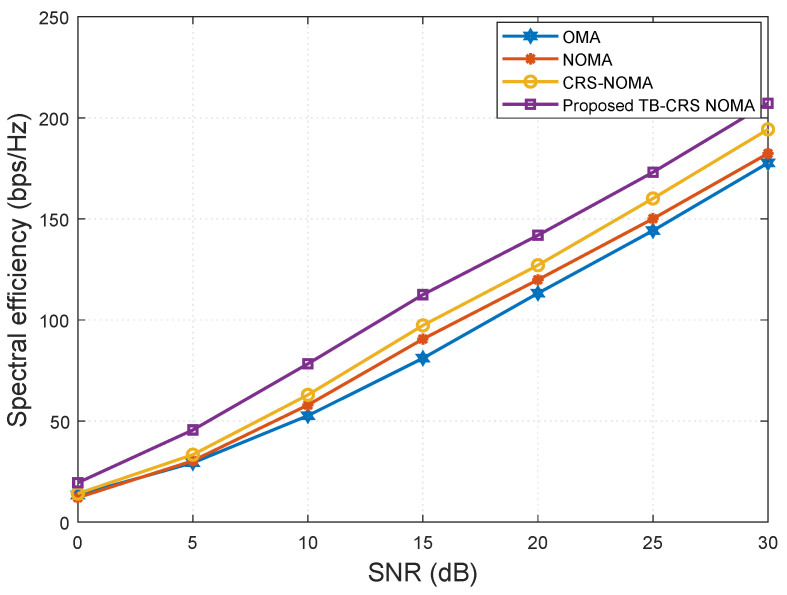
Spectral efficiency versus SNR for *K* = 32 MUTs.

Figure 6 shows the spectral efficiency versus SNR when the number of MUTs served by the gNB is K=32 and the SINR threshold is $\gamma_{th}=-2$ dB [50]. It is clear from Figure 6 that the proposed threshold-based user-assisted CRS beamspace mMIMO NOMA system achieves higher spectral efficiency than all the baseline systems. The proposed scheme shows a gain of approximately 15 bps/Hz at SNR = 10 dB compared with the CRS beamspace mMIMO NOMA system [3]. In addition, the proposed system shows a gain of approximately 25 bps/Hz and 30 bps/Hz at SNR = 10 dB compared with the beamspace MIMO-NOMA [4] and MIMO-OMA [49] systems, respectively, and the performance gap remains constant with increasing SNR.

**Figure 7:**
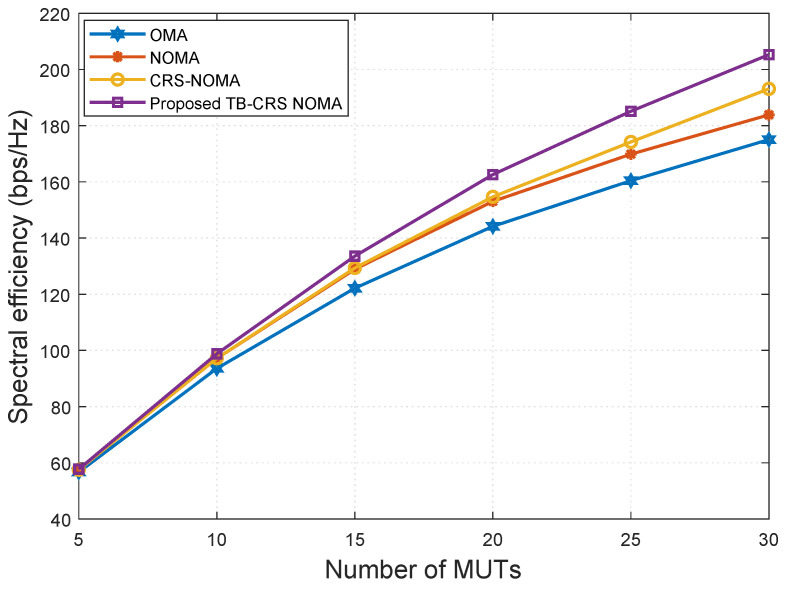
Spectral efficiency versus the number of MUTs when SNR = 20 dB.

Figure 7, plotted using (35), shows a comparison of the spectral efficiency versus the number of MUTs of the proposed system and the baseline systems, where SNR is 20 dB, the SINR threshold is $\gamma_{th}=-2$ dB [50], and the number of MUTs is increased from 5 to 30. It can be inferred from this figure that the spectral efficiency of the proposed system is higher than that of the other three systems [3,4,49]. As the number of MUTs increases, the performance gap between the proposed threshold-based user-assisted CRS and the CRS beamspace mMIMO NOMA system [3] increases monotonically. This is because, as the number of MUTs increases, the probability of different MUTs selecting the same beam becomes very high [11]. Consequently, the number of time slots required by the CRS beamspace mMIMO NOMA system [3] to deliver the symbols of the cell-edge MUT will increase exponentially with the number of MUTs within the NOMA cluster, whereas the cell-edge MUT is required to receive all symbols within a fixed time frame. Meanwhile, the beamspace MIMO-NOMA [4] and MIMO-OMA systems [49] show inferior performance and, in general, the MIMO-OMA system has the lowest performance.

### 4.2. Energy Efficiency

The energy efficiency $\epsilon_{EE}$ is the ratio of the feasible sum rate $C^{Total}$ to the total power consumption of the system [3,4] and can be expressed as

(40) $$\epsilon_{EE}=\frac{C^{Total}}{P_{t}+N_{RF}P_{RF}+N_{RF}P_{SW}+P_{BB}},$$

where $P_{t}$ is the total transmit power, $P_{RF}$ is the power exhausted at each RF block, $P_{SW}$ is the power utilized by each switch, and $P_{BB}$ is the power utilized at the baseband [3,4]. The values adopted for this simulation are $P_{t}=32$ mW, $P_{RF}=300$ mW, $P_{SW}=5$ mW, and $P_{BB}=200$ mW [3,4].

**Figure 8:**
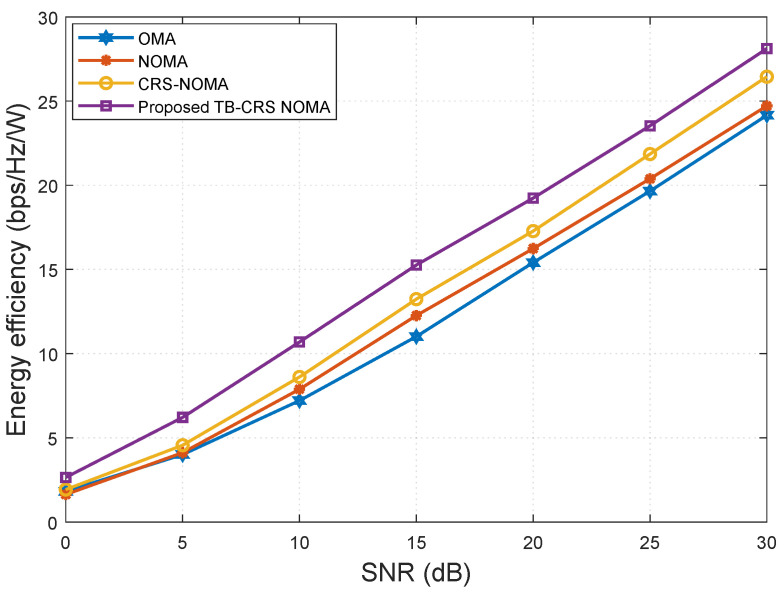
Energy efficiency versus SNR for *K* = 32 MUTs.

In Figure 8, the energy efficiency versus SNR of the proposed system is compared with that of the baseline systems [3,4,49] for K=32 and SINR threshold $\gamma_{th}=-2$ dB [50]. From this figure, it can be concluded that the proposed threshold-based user-assisted CRS beamspace mMIMO NOMA outperforms the baseline systems [3,4,49]. In particular, the proposed system shows a gain of approximately 2.5 bps/Hz/W at SNR = 10 dB compared with the CRS beamspace mMIMO NOMA system [3]. The performance gap between the proposed system and the CRS beamspace mMIMO NOMA system [3] remains constant as the SNR increases. In addition, the proposed threshold-based user-assisted CRS beamspace mMIMO NOMA also outperforms beamspace MIMO-NOMA [4] and MIMO-OMA [49], showing gains of approximately 3 bps/Hz/W and 4 bps/Hz/W, respectively, at SNR = 10 dB. Moreover, our proposed system only requires two transmission slots to achieve cooperative transmission regardless of the number of MUTs, whereas for the CRS beamspace mMIMO NOMA system [3], the number of required transmission slots increases linearly with the number of MUTs. This advantage is a result of utilizing NOMA, MRC, and the proposed threshold-based relaying strategy.

**Figure 9:**
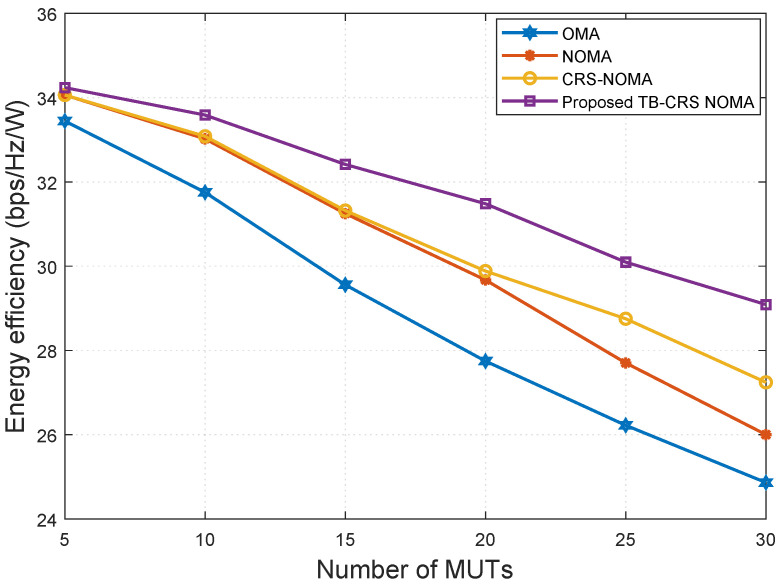
Energy efficiency versus the number of MUTs when SNR = 20 dB.

Figure 9 shows the energy efficiency versus the number of MUTs when SNR is 20 dB and the SINR threshold is $\gamma_{th}=-2$ dB [50]. The energy efficiency of the proposed system is higher than that of the baseline systems even when the number of MUTs is very large. All schemes show decreasing energy efficiency as the number of MUTs increases, but the proposed method continues to show the highest performance.

### 4.3. Outage Probability

**Figure 10:**
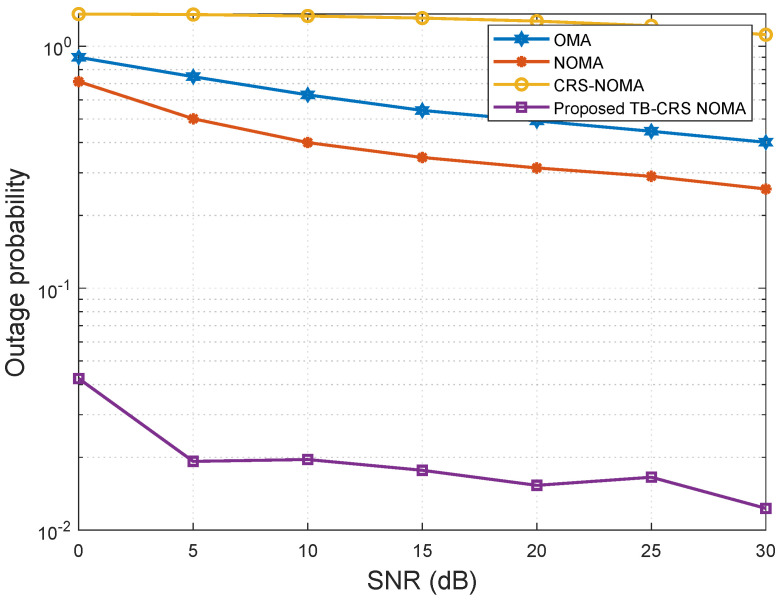
Outage probability versus SNR for *K* = 32 MUTs.

Figure 10, plotted using (38), shows the outage probability of the cell-edge MUT versus SNR when the minimum data rate is $R_{min}=0.5$ bps/Hz and the SINR threshold is $\gamma_{th}=-2$ dB [50]. The analytic curves for the outage probability of the cell-edge MUT in the CRS beamspace mMIMO NOMA [3], beamspace MIMO-NOMA [4], and MIMO-OMA [49] systems and the proposed threshold-based user-assisted CRS beamspace mMIMO NOMA system are plotted. As can be seen in Figure 10, the cell-edge MUT of the proposed CRS beamspace mMIMO NOMA system has a lower outage probability than the existing CRS beamspace mMIMO NOMA system [3]. This is because an outage event at any relaying MUT (i.e., any relay within the multi-hop) in the CRS beamspace mMIMO NOMA system results in an outage at the cell-edge MUT. However, in the proposed threshold-based user-assisted CRS beamspace mMIMO NOMA, an outage event occurs at the cell-edge MUT only when there is an outage at the direct link from the gNB and all the links from the relaying MUTs. Moreover, the proposed threshold-based user-assisted CRS beamspace mMIMO NOMA system has a lower outage probability than the beamspace MIMO-NOMA [4] and MIMO-OMA [49] systems. The existing CRS beamspace mMIMO NOMA [3] system has the lowest outage probability performance.

**Figure 11:**
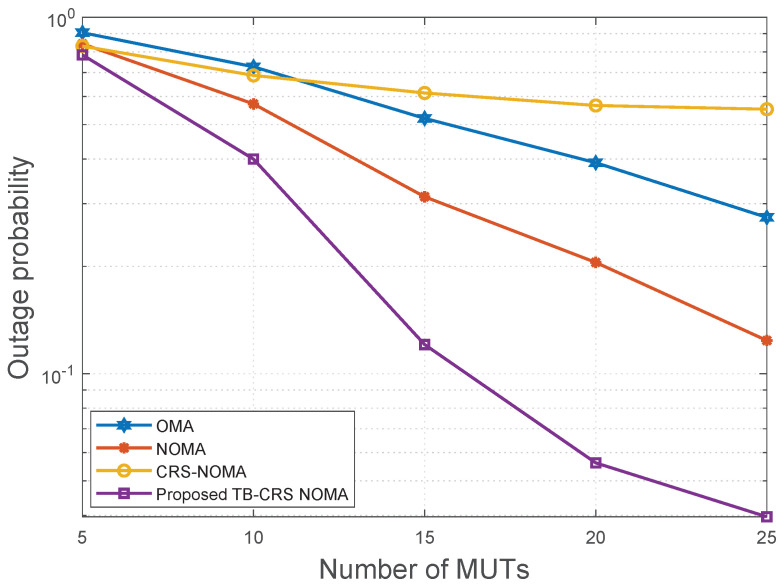
Outage probability versus the number of MUTs when SNR = 20 dB.

Figure 11 shows the outage probability of the cell-edge MUT versus the number of MUTs when the minimum data rate is $R_{min}=0.5$ bps/Hz, SNR is 20 dB, and the SINR threshold is $\gamma_{th}=-2$ dB [50]. When there are only 5 MUTs served by the gNB, the outage probability of the proposed threshold-based user-assisted CRS beamspace mMIMO NOMA system has a slightly lower outage probability than the CRS beamspace mMIMO NOMA [3], beamspace MIMO-NOMA [4], and MIMO-OMA [49] systems. This is because the fewer the number of MUTs, the lower the probability of different MUTs selecting the same beam [11]. Moreover, the performance of CRS beamspace mMIMO NOMA [3] and beamspace MIMO-NOMA [4] is similar, while MIMO-OMA [49] shows the worst performance. As the number of MUT increases, the outage probability of the proposed threshold-based user-assisted CRS beamspace mMIMO NOMA system decreases further and continues to show better performance than the baseline systems. In addition, the CRS beamspace mMIMO NOMA [3] has the worst performance among the baseline systems when the number of MUTs becomes greater than 12. This is because, as the number of MUTs increases, the number of MUTs sharing the same beam also increases, resulting in more time slots required to transmit the signal of the cell-edge MUT while the cell-edge MUT is delay-intolerant. As the number of MUTs increases further, the proposed threshold-based user-assisted CRS beamspace mMIMO NOMA system continues to show superior performance compared to all the baseline systems.

## 5. Conclusions

We proposed threshold-based user-assisted cooperative relaying in a beamspace mMIMO NOMA system that exhibits a feasible sum rate for *K* MUTs in mmWave communications. This proposed system was applied in a dynamically formed group of NOMA MUTs, where the number of MUTs within the beam exceeds one. In particular, the MUTs within a NOMA group closer to the gNB relay symbols that are intended for the cell-edge MUT after successful SIC only when they meet a predetermined SINR threshold requirement in order to balance between the number of relay MUTs and the system performance. ZF precoding and an iterative power allocation were utilized to maximize the system sum rate and minimize intra- and inter-beam interferences. The effectiveness of the proposed threshold-based user-assisted CRS beamspace mMIMO NOMA was confirmed by computer simulation results, which revealed that the proposed strategy can achieve higher performance than CRS beamspace mMIMO NOMA, beamspace MIMO-NOMA, and MIMO-OMA systems in terms of spectral efficiency, energy efficiency, and outage probability. Moreover, the proposed system can be utilized to extend the coverage area and guarantee reliable transmission in mmWave communications. We intend to investigate channel estimation in threshold-based user-assisted cooperative relaying in a beamspace mMIMO NOMA in the future.

## Figures and Tables

**Table 1:** A list of the distinctions between the suggested method and some relevant literature.

	[36]	[43]	[37]	[40]	[41]	[32]	Current work
Channel	AGN	QR + LS	QR	Discrete AGN	AGN	mmWave	mmWave
Uplink							
Downlink	✓	✓	✓	✓	✓	✓	✓
D2D							
Dedicated relay	✓		✓	Partial	Partial		
User-assisted CRS		✓				✓	✓
Duplex mode	Half	Full/Half	Half	Half	Half	Half	Half
Decode-and-forw.		✓	✓	✓	✓	✓	✓
Amplify-and-forw.	✓						
Multi-hop						✓	
Multi-relay		✓	✓		✓	≥1 per beam	≥1 per beam
Single-relay	✓			✓			
Destination	1	2	2	1	1	1 per beam	1 per beam
Combining	2 MRC	SC					MRC
SIC	2	✓	✓	Relay	Relay	Relay	Relay & C-MUT
Threshold-based							✓
Buffer-aided			✓				
Outage probability	✓	✓					✓
Sum rate					Rate region	✓	✓
Energy efficiency						✓	✓
Throughput		✓	✓	✓			
Ergodic capacity	✓	✓					
Multiple access	NOMA	NOMA	NOMA/OMA			NOMA	NOMA
Message splitting				✓			
Precoding				GDPC		ZF	ZF
Time slot	2	≥1	2 + buffer delay	2	2	No. of MUTs	2

## Data Availability

Not applicable.

## Abbreviations

The following abbreviations are used in this manuscript:

AGN	Additive Gaussian noise
GDPC	Generalize dirty paper coding
C-MUT	Cell-edge mobile user terminal
MRC	Maximal ratio combining
SC	Selection combining
QR	Quasi-static Rayleigh fading
LS	Large-scale path loss
